# Verification of surface electromyographic activity of the oblique externus abdominis using ultrasound shear wave elastography

**DOI:** 10.14814/phy2.15295

**Published:** 2022-05-05

**Authors:** Kentaro Chino, Ryosuke Ando, Yasuhiro Suzuki

**Affiliations:** ^1^ 12892 Department of Health and Physical Education Faculty of Human Development Kokugakuin University Kanagawa Japan; ^2^ Sports Okinawa Okinawa Japan; ^3^ 105249 Department of Sports Research Japan Institute of Sports Sciences Tokyo Japan; ^4^ 52766 Center for General Education Tokyo Keizai University Tokyo Japan

**Keywords:** abdominal muscles, oblique internus abdominis, signal crosstalk, surface electromyography, trunk rotation

## Abstract

This study used ultrasound shear wave elastography (SWE) to revalidate whether surface electromyographic (EMG) electrodes placed on the oblique externus abdominis (OE) can detect only the OE activity without the confounding activity of the underlying oblique internus abdominis (OI). During left and right trunk rotations, the EMG activity was acquired using surface EMG electrodes placed on the right OE. Shear wave velocity (V_s_) values of the right OE and OI were acquired using SWE. The EMG activity during the left and right trunk rotations significantly increased as the level of exertion increased (25%, 50%, 75%, and 100% of the one‐repetition maximum [1RM]). The V_s_ of the right OE was significantly different only between 25% and 75% 1RM in the right trunk rotation, but significantly increased from 25% to 75% 1RM during the left trunk rotation. The V_s_ of the right OI during the right trunk rotation significantly increased with increased levels of exertion, except between 50% and 75% 1RM. The results for the V_s_ of the OE and OI in the right trunk rotation suggest that surface EMG electrodes placed on the OE would detect not only the antagonistic OE activity but also the agonistic OI activity.

## Graphical abstract

Ultrasound shear elastography (SWE) can quantify the activity of the oblique externus abdominis (OE) and the oblique internus abdominis (OI) separately without one muscle influencing the other. Therefore, this study used ultrasound shear wave elastography (SWE) to revalidate whether surface electromyographic (EMG) electrodes placed on the OE can detect only the OE activity without the confounding activity of the underlying OI. The findings on ultrasound SWE obtained in this study led to the conclusion that surface EMG electrodes placed on the OE appear to detect not only the activity of the OE, but also that of the underlying OI.

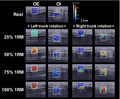

## 1 INTRODUCTION

**FIGURE 1:**
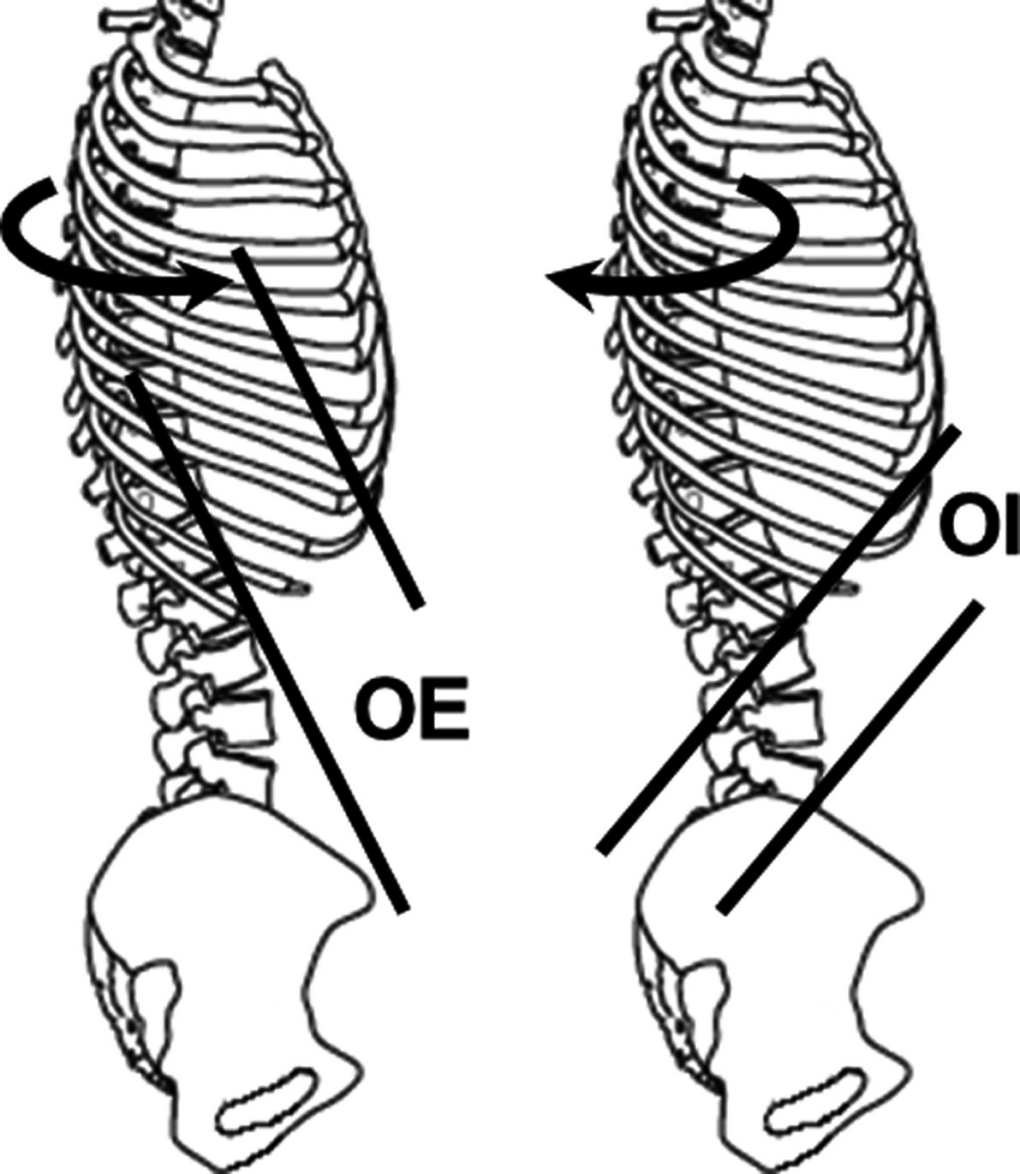
Schematic representation of the average fiber direction of the oblique externus abdominis (OE) and oblique internus abdominis (OI) muscles, and the direction of trunk rotation. The average fiber directions of the OE and OI are drawn based on those reported by Brown et al. (2011). The fiber direction of the OE is nearly perpendicular to that of the underlying OI. Therefore, unilateral contractions of the OE and OI lead to trunk rotation in the opposite directions: The right OE produces trunk rotation to the left, while the right OI produces trunk rotation to the right

The oblique externus abdominis (OE) is a lateral abdominal muscle that is the most effective axial rotator of the trunk (Neumann, 2017). The OE is the most superficial of the lateral abdominal muscles; therefore, previous studies have measured its activity using surface electromyography (EMG) (Boccia & Rainoldi, 2014; McGill et al., 1996; Ng et al., 2001). McGill et al. (1996) measured the OE activity not only by using surface electrodes placed on the OE, but also by using intramuscular electrodes inserted into the OE. The differences in the root mean square (RMS) value between the surface and intramuscular electrodes were 11% of maximum voluntary contraction (MVC) during torso flexion, 5% of MVC during torso extension, 8% of MVC during lateral bend, and 10% of MVC during twisting. They considered that an RMS difference within 10% of MVC was reasonable and acceptable to assist clinical interpretations of the EMG amplitude, and ultimately for modeled estimates of muscle force; therefore, they concluded that surface EMG adequately represents the OE activity. However, the authors did not examine the OE activity during trunk rotation, where the OE acts as the antagonist and the oblique internus abdominis (OI) acts as the agonist. The OI is located immediately beneath the OE, forming the second layer of the lateral abdominal muscles, and is the most effective axial rotator of the trunk along with the OE (Neumann, 2017). However, the OI, unlike the OE, is not a contralateral axial rotator, but an ipsilateral axial rotator: Unilateral contraction of the OI leads to ipsilateral trunk rotation (Brown et al., 2011; Neumann, 2017; Ng et al., 2001) (Figure 1). Hence, the OE and OI are classified as either a prime mover or an antagonist depending on the direction of axial rotation (Ng et al., 2001): The OE works synergistically with the contralateral OI as the agonist for axial rotation. Considering the location of the OE and OI and their role in trunk rotation, it is possible that the surface electrodes placed on the OE detect not only the antagonistic OE activity but also the agonistic OI activity during the trunk rotation. To examine whether the surface electrodes placed on the OE measure the activity of the OE without the confounding activity of the underlying OI, measurements for trunk rotation where the OI is significantly activated as the agonist are required.

Ultrasound shear wave elastography (SWE) is an ultrasound‐based imaging technique that can quantitatively assess localized tissue elasticity *in vivo* (Bercoff et al., 2004; Gennisson et al., 2013; Hug et al., 2015). Previous studies have used ultrasound SWE for measurements of the OE and OI, confirming the reliability of the measurement at rest (Linek et al., 2019; MacDonald et al., 2016), during abdominal hollowing and bracing (MacDonald et al., 2016), and during asymmetrical isometric muscle contraction (Linek et al., 2019). In this technique, an acoustic radiation force is created by focusing an ultrasonic beam deep into the tissues, and remotely generating shear waves that propagate transversely to the radiation force. Shear wave velocity (V_s_) is directly related to the shear elastic modulus (μ) by the elastic wave equation: μ = ρV_s_^2^, where ρ is the density of the tissues. Additionally, V_s_ is highly related to the levels of muscle activity (Lapole et al., 2015; Nordez & Hug, 2010; Yoshitake et al., 2014). Thus, the V_s_ acquired using ultrasound SWE could be used as an indicator of muscle activity without the influence of the confounding activity of the adjacent muscles. Ultimately, ultrasound SWE offers an advantage over surface EMG, as it can reliably quantify the activity of the OE and OI separately without one muscle influencing the other. Hence, we used ultrasound SWE to revalidate measurements of the OE activity using surface EMG. More specifically, we aimed to examine whether surface EMG could measure the activity of the OE without including the confounding activity of the underlying OI, especially during trunk rotation where the OE acts as the antagonist and the OI acts as the agonist.

## 2 MATERIALS AND METHODS

### 2.1 Participants

Ten healthy men (mean ± standard deviation [SD]: Age, 20.7 ± 0.8 years; height, 172.4 ± 3.1 cm; body mass, 65.4 ± 6.5 kg) without any history or clinical signs of orthopedic or neuromuscular disorders participated in this study. Each participant was informed about the purpose of this study and the experimental protocol before providing written informed consent to participate. This study was approved by the ethical review board of Kokugakuin University (R01 No. 6) and was conducted in accordance with the tenets of the Declaration of Helsinki and its subsequent amendments.

### 2.2 Experimental setup

**FIGURE 2:**
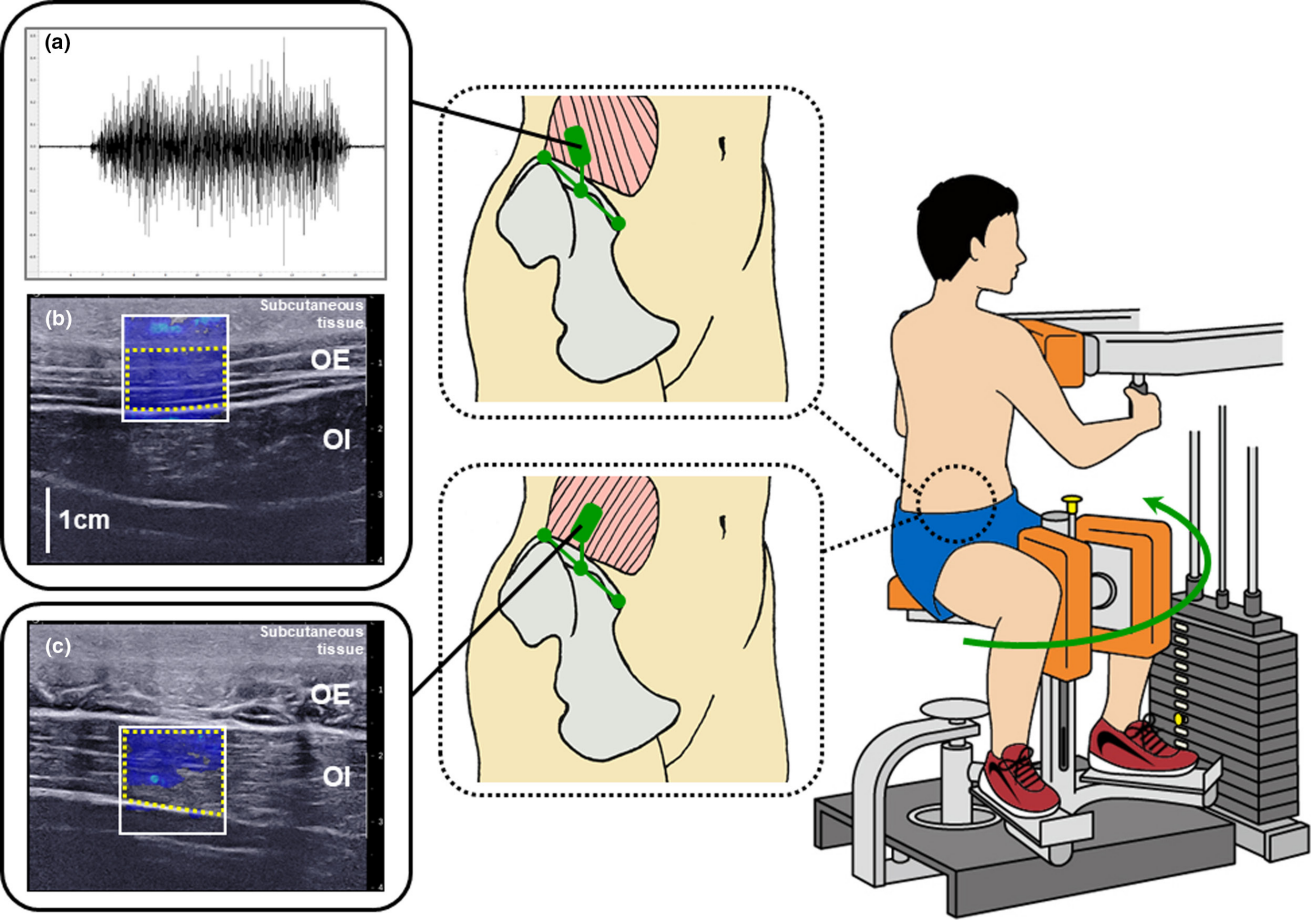
Experimental setup for muscle activity measurements by (a) surface electromyography (EMG) and (b and c) ultrasound shear wave elastography (SWE). The illustration shows the placement of the surface EMG sensor and the ultrasound probe at the same site on the skin surface. Participants faced forward with their lower bodies rotated on a swivel to the right or left while maintaining the position for 5 s against the weight. A 1.5 × 1.5 cm region of interest for ultrasound SWE indicated by the white square was placed on the oblique externus abdominis (OE) or oblique internus abdominis (OI), and the average shear wave velocity of the OE and OI indicated by the yellow dotted line was quantified

A weight‐stack rotary torso and twist training machine (FUNASIS BB4820 Rotary Torso & Twist; Senoh Corporation, Chiba, Japan) was used to perform trunk rotations (Figure 2). This machine was stocked with 1.5–53.5 kg weight‐stack adapter plates, and the weight could be adjusted in increments of 2.6 kg. In the trunk rotation, the participants placed their feet on the swivel with their upper bodies pressed against the upper pad.

Surface EMG signals were acquired using a surface EMG sensor with a pre‐amplifier (FAD‐SEMG1; 4Assist, Tokyo, Japan). The signals were differentially sampled by two silver bar electrodes (0.1 × 0.5 cm each) with a 1‐cm interelectrode distance and pre‐amplification (gain, 500‐fold; frequency response, 5–1000 Hz; input impedance, 100 GΩ; common‐mode rejection ratio, 80 dB). Prior to attaching the sensor, the skin surface was cleaned with alcohol and rubbed with sand particles. The sensor was placed on the skin over the right OE, according to the insertion point of intramuscular EMG described by Perotto (2011); midway along the line between the highest point of the iliac crest and the anterior superior iliac spine, with the sensor placed just cephalad to the iliac crest (Figure 2). To facilitate placement of the sensor along the fascicle direction of the OE, the direction was detected using an ultrasound apparatus (Aixplorer; Supersonic Imagine, Aix‐en‐Provence, France) coupled with a linear array probe (4–15 MHz, SuperLinear 15–4; Vermon, Tours, France) in B‐mode. The reference electrode was wrapped around the right wrist. The amplified EMG signals were converted using an analog‐to‐digital converter (PowerLab 16/35; AD Instruments, New South Wale, Australia) at a sampling rate of 1000 Hz and stored on a personal computer.

Color‐coded quantitative elasticity maps were acquired at a sampling rate of 1 Hz using an ultrasound apparatus (Aixplorer; Supersonic Imagine, Aix‐en‐Provence, France) with a linear array probe (4–15 MHz, SuperLinear 15–4; Vermon, Tours, France) in SWE mode (Figure 2). To ensure that the elasticity maps were acquired from the same site on the skin surface where the EMG signals were acquired, the skin surface where the EMG sensor and ultrasound probe were placed was marked by a felt‐tipped pen. The fascicle direction of the OE was detected using the ultrasound apparatus in B‐mode, and the principal axis of the probe was placed along the fascicle direction. Because the average fiber direction of the OI is nearly perpendicular to the direction of the OE (Neumann, 2017) (Figure 1), elasticity maps along the fascicle direction of the OI were acquired separately from the elasticity maps of the OE. In order to avoid excessive compression of the skin surface during the measurements, the probe was coated with a transmission gel, and the examiner, who had 18 years of experience in musculoskeletal ultrasound measurements, controlled probe compression during the measurements.

### 2.3 Experimental protocol

From the starting position where the trunk was rotated 50° to the right or left, the participants faced forward by rotating their lower body on the swivel to the left or right and held a front‐facing position (i.e., the neutral rotational position) for 5 s (Figure 2). The one‐repetition maximum (1RM) was defined as the maximum weight that the participants could lift while maintaining the front‐facing position for 5 s; the 1RM was measured separately for the left and right trunk rotations. The weight in the trunk rotation was set at 25%, 50%, 75%, and 100% 1RM. Because the EMG activity, elasticity map of the OE, and elasticity map of the OI were acquired separately, not simultaneously, trunk rotations at each weight were performed six times (twice for each of the 3 measurements). To exclude the confounding influence of fatigue, the measurements of the EMG activity and elasticity map of the OE and OI were administered in a random order, and rest periods of at least 1 min were permitted between the trunk rotations.

### 2.4 Data analysis

Electromyography signals were processed using data analysis software (LabChart Pro v8.1.9; ADInstruments, New South Wale, Australia). A bandpass filter at 5–500 Hz was applied to the EMG signals (Merletti, 1999), followed by calculation of the RMS amplitude of the EMG signals over 4 s during the 5‐s trunk rotation. Because the highest RMS amplitude was acquired during the left trunk rotation at 100% 1RM in all the participants, the RMS amplitude during each rotation was normalized to the amplitude observed during the left trunk rotation at 100% 1RM.

Since the trunk rotation was maintained for 5 s and the sampling rate of the SWE was 1 Hz, five elasticity maps could be acquired; the fifth elasticity map was adapted and used for further analysis. Using the built‐in software of the ultrasound apparatus, the average V_s_ values of the OE and OI were evaluated by tracing the areas where these muscles were seen in a 1.5 × 1.5 cm region of interest for the elasticity maps (Figure 2). The elasticity maps of the OE and OI at rest were acquired in the front‐facing position, and the V_s_ at rest of these muscles were also evaluated. The total thickness of the subcutaneous tissue and the OE overlying the OI (distance between the ultrasound probe‐skin surface and the OE‐OI interface) at rest was measured from the B‐mode ultrasound image displayed behind each elasticity map (Figure 2).

### 2.5 Statistics

With respect to the EMG amplitude and V_s_, post hoc power analysis for two‐tailed matched pairs *t*‐test was conducted. Statistical power (1 – β error probability), the probability of correctly rejecting the null hypothesis when it is false (Faul et al., 2007; Vincent, 2005), was computed as a function of significance level (α error probability), sample size, and population effect size. The significance level was set to 0.05, and the population effect size (Cohen's d_z_) was calculated by the following equation (Lakens, 2013):

$${\text{Cohen}}^{\prime }s\;d_{z}=\frac{{\text{Mean}}_{\text{diff}}}{\sqrt{\frac{\sum {\left(X_{\text{diff,i}}‐{\text{Mean}}_{\text{diff}}\right)}^{2}}{n‐1}}},$$

where Mean_diff_ is the difference between the means, X_diff, i_ is the difference between the two measurements of each participant, and n is the number of participants. When comparing the V_s_ of the OI at rest (n_1_ = 5) with the V_s_ of the OI at 25%–100% 1RM (n_2_ = 10), the following effect size (Cohen's d_s_) was used to consider the difference in the number of participants (Lakens, 2013):

$${\text{Cohen}}^{\prime }s\;d_{s}=\frac{{\text{Mean}}_{\text{diff}}}{\sqrt{\frac{\left(n_{1}‐1\right){\text{SD}}_{1}^{2}+\left(n_{2}‐1\right){\text{SD}}_{2}^{2}}{n_{1}+n_{2}‐2}}},$$

where SD is the standard deviation of each measurement. When computing 1 – β using the Cohen's d_s_, the sample size was not set to 10, but to 5. Because the convention proposed for general use of statistical power is 1 – β = 0.8 (Cohen, 1992), when 1 – β was greater than 0.8, it was considered that a significant difference had been correctly detected. The power analysis was performed using GPower 3.1.9.7, a stand‐alone power analysis program (Erdfelder et al., 1996; Faul et al., 2007, 2009). All data for the EMG amplitude and V_s_ obtained from each participant were pooled, and the correlation between the EMG amplitude and V_s_ was determined by Pearson's correlation coefficient. Before determining the correlation, the V_s_ of the OE at each level of exertion was normalized to the V_s_ of the OE in the left trunk rotation at 100% 1RM, and the V_s_ of the OI at each level of exertion was normalized to the V_s_ of the OI in the right trunk rotation at 100% 1RM. The level of statistical significance was set at *p* < 0.05. All the data are expressed as mean ± SD.

## 3 RESULTS

**FIGURE 3:**
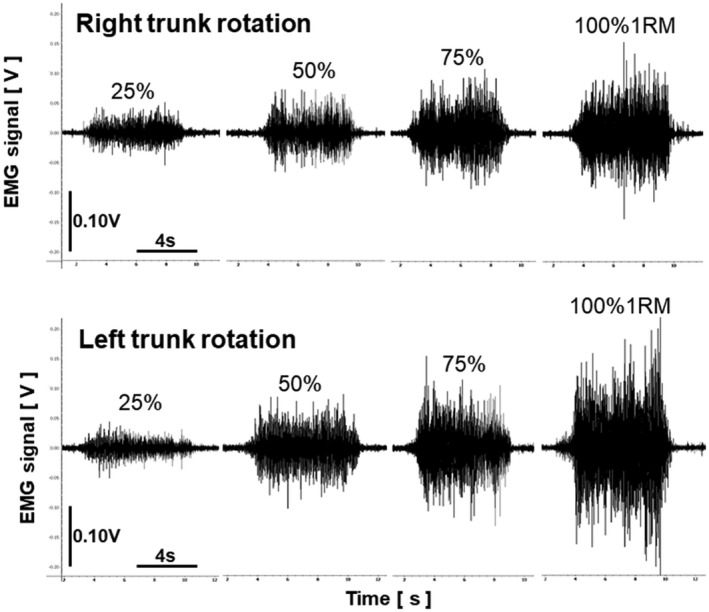
Typical surface electromyographic (EMG) signals during right (top) and left (bottom) trunk rotations are acquired using surface EMG electrodes placed on the right oblique externus abdominis. The loads during the trunk rotation were set at 25%, 50%, 75%, and 100% of the one‐repetition maximum (1RM)

**FIGURE 4:**
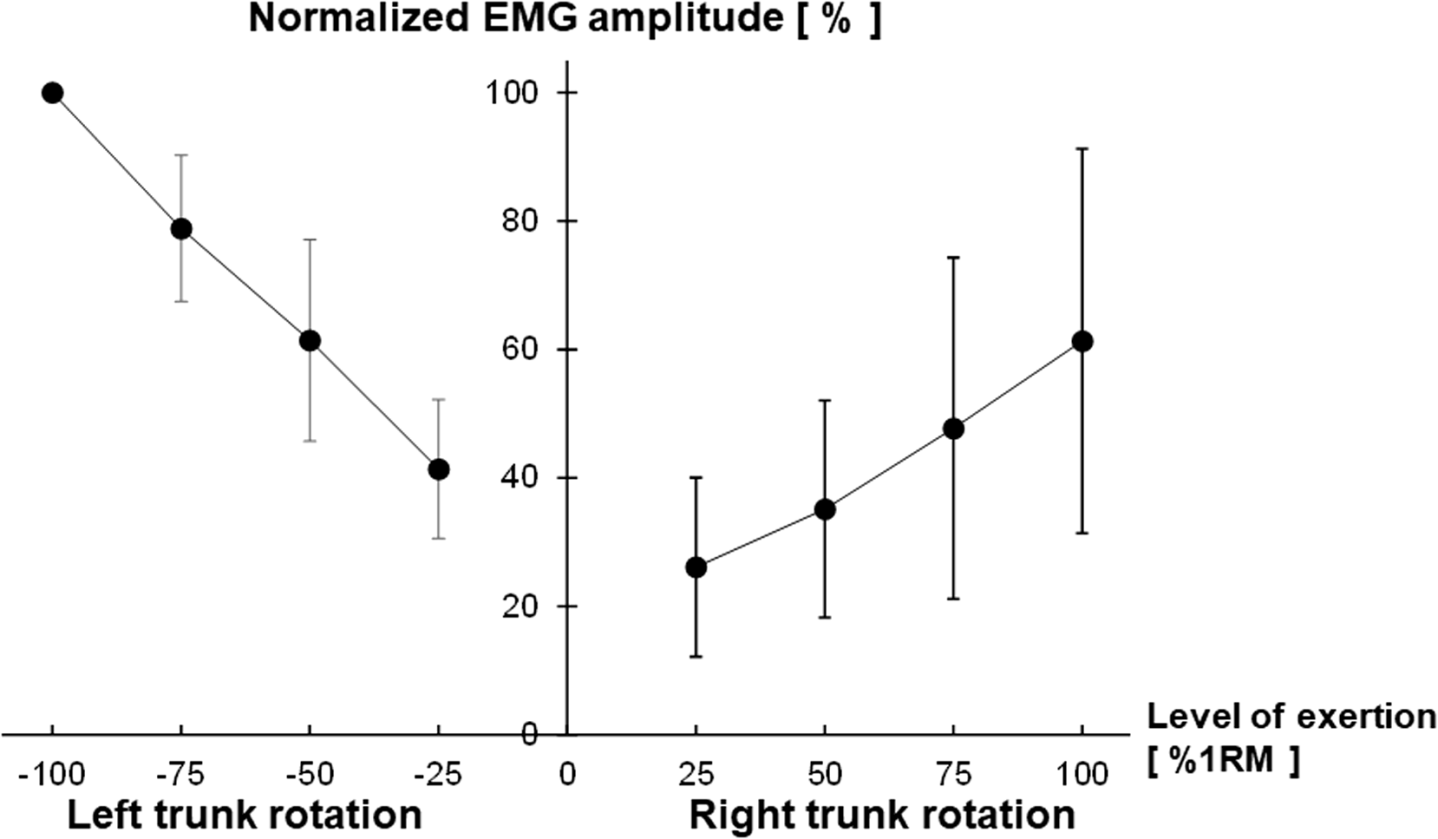
Relationship between the level of exertion and the surface electromyographic (EMG) amplitude in left and right trunk rotations. The levels of exertion shown are equivalent to 25%, 50%, 75%, and 100% of the one‐repetition maximum (1RM). The root mean square amplitudes of the EMG signals during each trunk rotation are normalized to the amplitude during the left trunk rotation at 100% 1RM. The EMG amplitudes of both the left and right trunk rotations were significantly different for all combinations of the exertion levels

**FIGURE 5:**
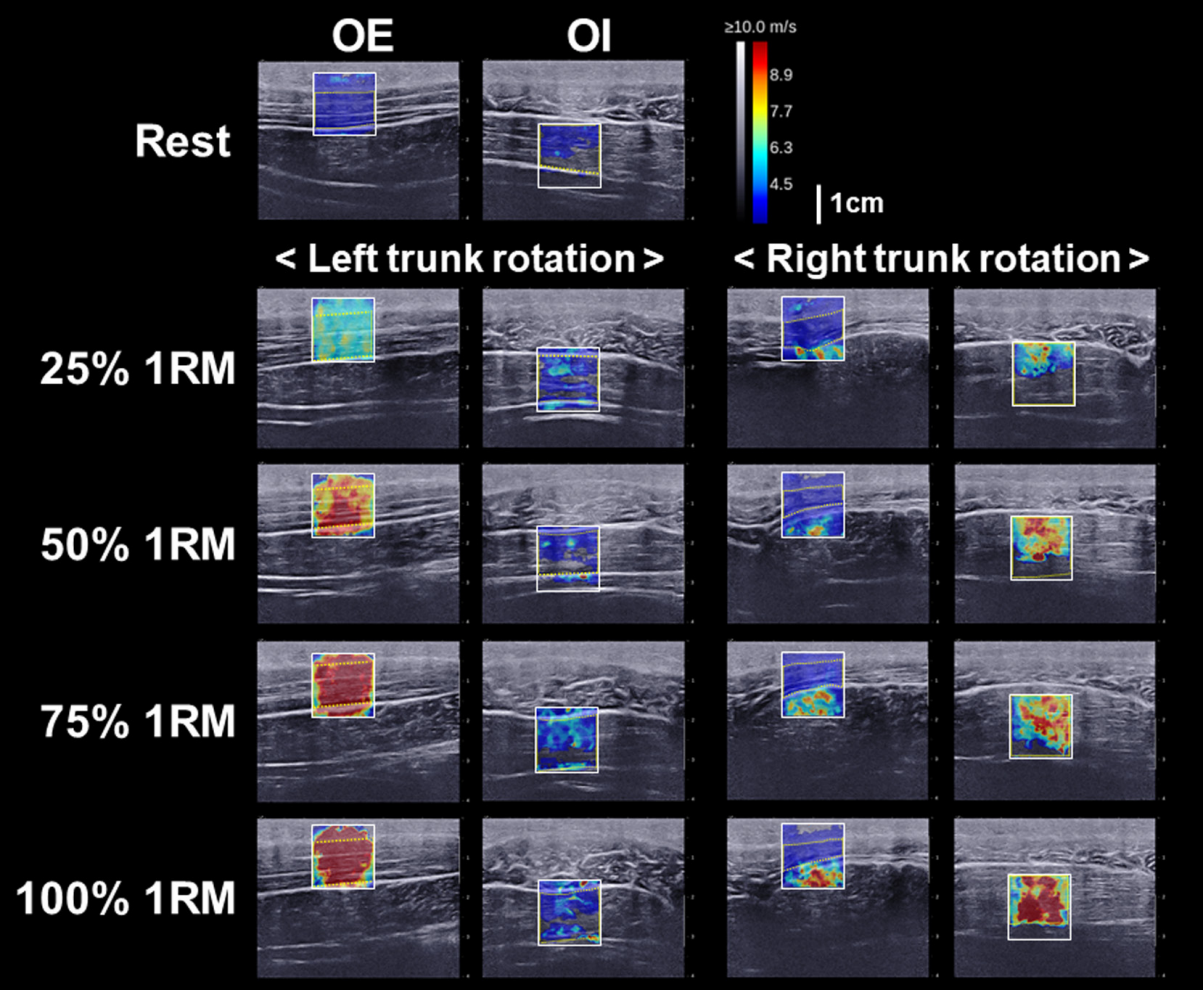
Typical color‐coded elasticity maps of the right oblique externus abdominis (OE) and right oblique internus abdominis (OI) during left and right trunk rotations. The elasticity maps were acquired using ultrasound shear wave elastography (SWE) at rest and during left and right trunk rotations at 25%, 50%, 75%, and 100% of the one‐repetition maximum (1RM). The 1.5 × 1.5 cm white squares indicate the region of interest for ultrasound SWE, and the yellow dotted line indicates the area where the average shear wave velocity of the OE and OI was quantified

**FIGURE 6:**
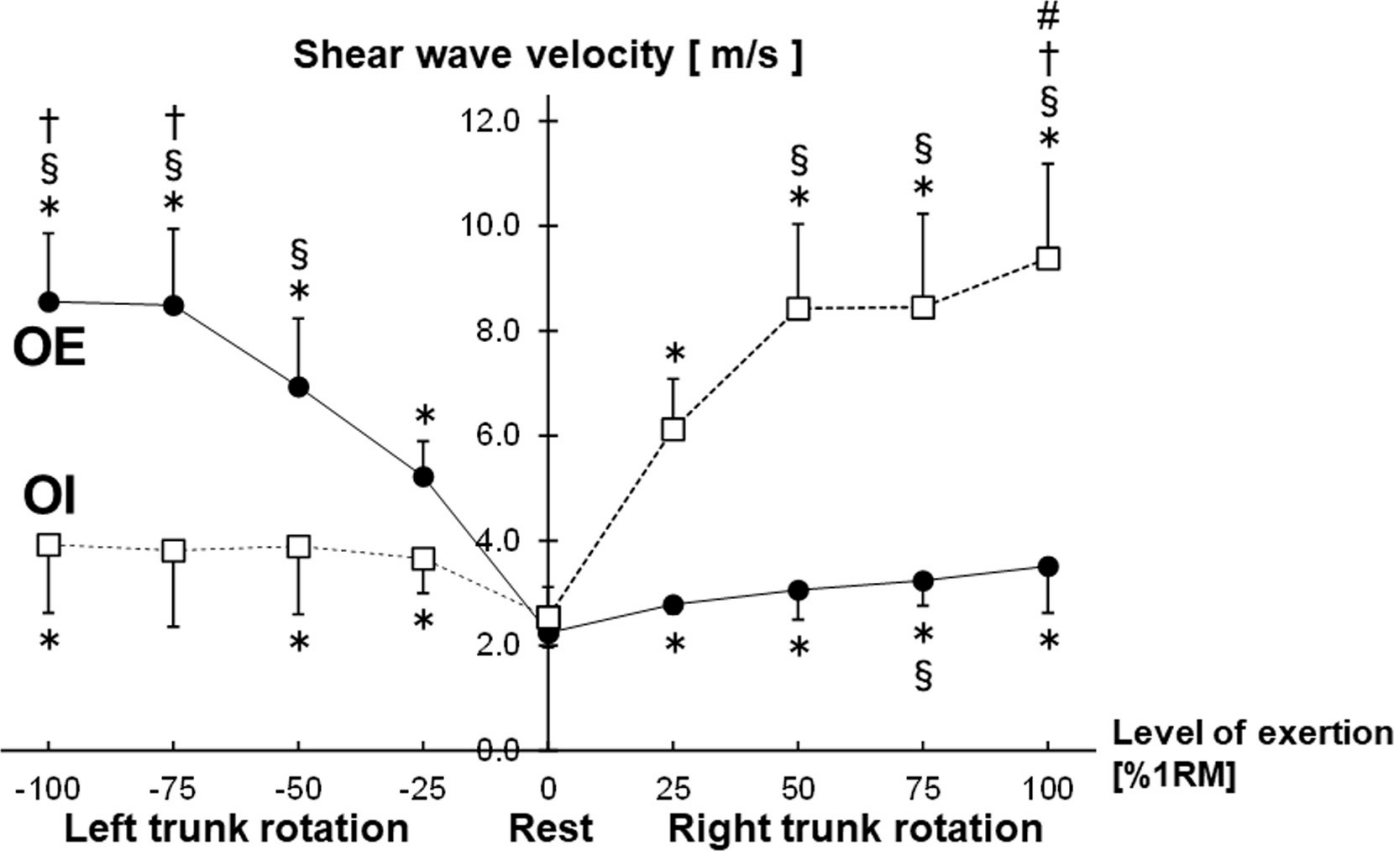
Relationship between the level of exertion and shear wave velocity in left and right trunk rotations. The levels of exertion shown are 0%, 25%, 50%, 75%, and 100% of the one‐repetition maximum (1RM). ^*^Statistically significant for 0% 1RM (i.e., at rest). ^§^Statistically significant for 25% 1RM. ^†^Statistically significant for 50% 1RM. ^#^Statistically significant for 75% 1RM

**FIGURE 7:**
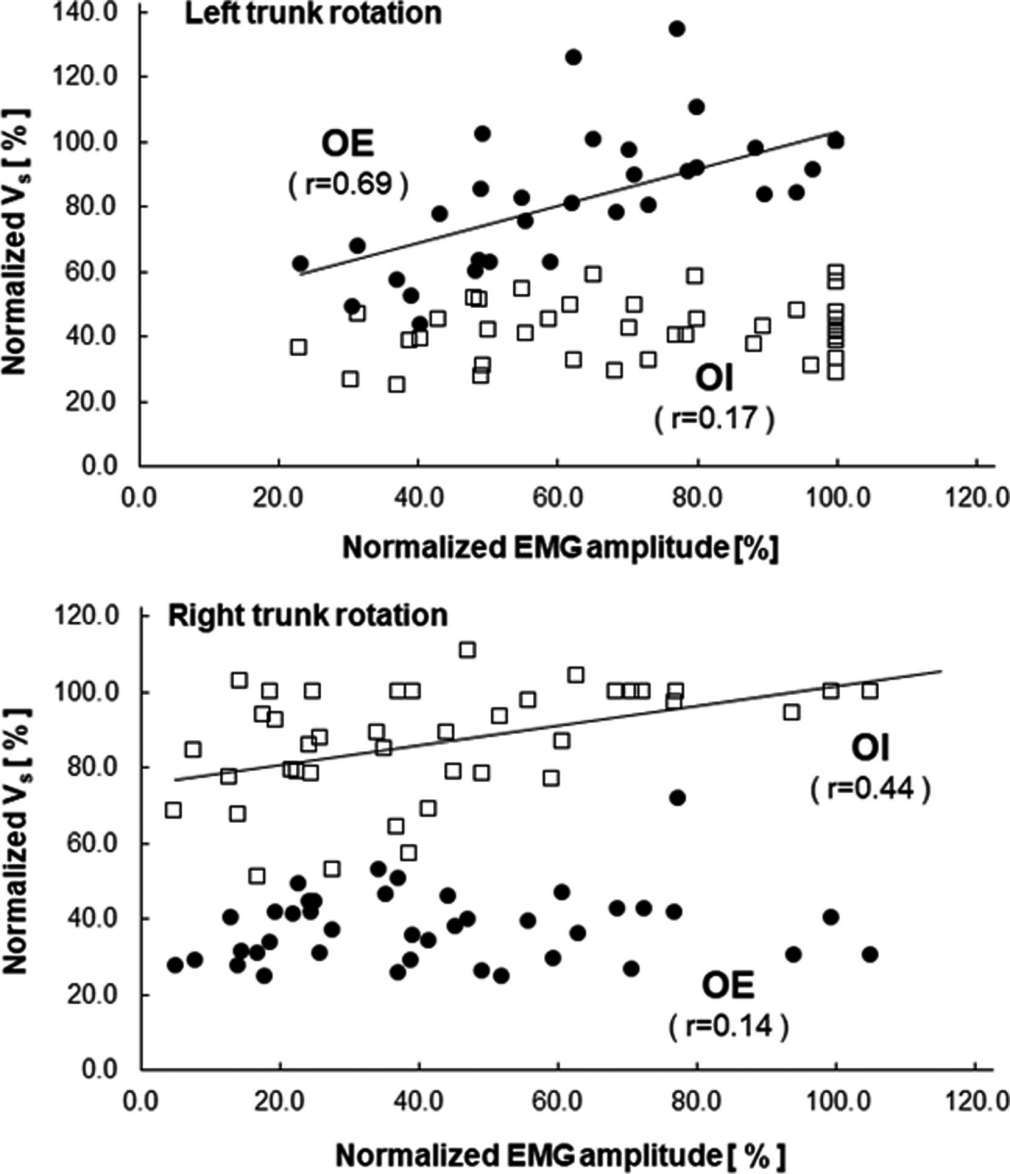
Relationship between the EMG amplitude and shear wave velocity (V_s_) of the oblique externus abdominis (OE) and oblique internus abdominis (OI). The data obtained from all participants (*n* = 10) are plotted together. The EMG amplitude at each level of exertion at 25%, 50%, 75%, and 100% 1RM was normalized to the amplitude during the left trunk rotation at 100% 1RM. The V_s_ of the OE was normalized to that of the OE during the left trunk rotation at 100% 1RM, and the V_s_ of the OI was normalized to that of the OI during the right trunk rotation at 100% 1RM. The EMG amplitude showed a significant correlation with the V_s_ of the OE during the left trunk rotation and the V_s_ of the OI during the right trunk rotation

Figure 3 shows the surface EMG signals during the left and right trunk rotations at 25%, 50%, 75%, and 100% 1RM. Clear EMG signals were observed during the left and right trunk rotations. Figure 4 shows the relationship between the level of exertion and EMG amplitude in the left and right trunk rotations. Post hoc power analysis revealed that the EMG amplitude was significantly different for all combinations of the exertion levels in the right trunk rotation (Cohen's d_z_ ≥ 1.03, 1 − β ≥ 0.83) and left trunk rotation (Cohen's d_z_ ≥ 1.58, 1 − β ≥ 0.99). The EMG amplitude at each level of exertion was significantly greater in the left trunk rotation compared to the right trunk rotation (Cohen's d_z_ ≥ 1.19, 1 − β ≥ 0.92). Figure 5 shows the B‐mode ultrasound image and the color‐coded elasticity maps of the OE and OI during trunk rotation. The total thickness of the subcutaneous tissue and the OE overlying the OI at rest was 1.8 ± 0.3 cm. The elasticity maps of the OI at rest could not be acquired for 5 of the 10 participants. During the left trunk rotation, an obvious change in the color of the elasticity map of the OE with the change in levels of exertion was observed, while no such obvious change was observed in the color of the elasticity map of the OI. On the other hand, during the right trunk rotation, an obvious change in the color of the elasticity map with the change in levels of exertion was observed only in the OI. Figure 6 shows the relationship between the level of exertion and V_s_ during the left and right trunk rotations. Post hoc power analysis revealed that the V_s_ of the OE during the left trunk rotation significantly differed among the levels of exertion at rest (0%), 25%, 50%, 75%, and 100% 1RM (Cohen's d_z_ ≥ 1.21, 1 − β ≥ 0.92), except between 75% and 100% 1RM (Cohen's d_z_ = 0.05, 1 − β = 0.05). The V_s_ of the OI during the left trunk rotation was significantly greater at 25%, 50%, and 100% 1RM than at rest (Cohen's d_s_ ≥ 1.70, 1 − β ≥ 0.81), whereas no significant differences were observed between at rest and 75% 1RM (Cohen's d_s_ = 1.64, 1 − β = 0.78) and among the levels of exertion at 25%–100% 1RM (Cohen's d_z_ ≤ 0.60, 1 − β ≤ 0.40). During the right trunk rotation, the V_s_ of the OE was significantly greater at 25%–100% 1RM than at rest (Cohen's d_z_ ≥ 1.35, 1 − β ≥ 0.97) and at 75% 1RM than at 25% 1RM (Cohen's d_z_ = 1.32, 1 − β = 0.96), but was not significantly different among the other paired comparisons of the level of exertion (Cohen's d_z_ ≤ 0.94, 1 − β ≤ 0.75). In the case of the OI, the V_s_ during the right trunk rotation was significantly different among the levels of exertion at 0%–100% 1RM (Cohen's d_z_ or d_s_ ≥ 1.17, 1 − β ≥ 0.91), except between 50% and 75% 1RM (Cohen's d_z_ = 0.02, 1 − β = 0.05). When comparing the V_s_ at each level of exertion, the V_s_ of the OE was significantly greater during the left trunk rotation than that during the right trunk rotation for all exertion levels (Cohen's d_z_ ≥ 2.55, 1 − β = 1.00). In contrast, the V_s_ of the OI was significantly greater during the right trunk rotation than that during the left trunk rotation at each level of exertion (Cohen's d_z_ ≥ 1.91, 1 − β = 1.00). The relationship between the EMG amplitude and V_s_ is shown in Figure 7. During the left trunk rotation, there was a significant correlation between the EMG amplitude and V_s_ of the OE (*r* = 0.69), but none between the EMG amplitude and V_s_ of the OI (*r* = 0.17). During the right trunk rotation, the correlation was not significant between the EMG amplitude and V_s_ of the OE (*r* = 0.14) but was significant between the EMG amplitude and V_s_ of the OI (*r* = 0.44).

## 4 DISCUSSION

This study acquired EMG signals during the left and right trunk rotations using the surface EMG electrodes placed on the right OE (Figure 2). The EMG signals were clearly observed during the left and right trunk rotations (Figure 3), and the EMG amplitude during the left and right trunk rotations significantly increased with an increase in the exertion level (Figure 4). These findings are consistent with those of Ng et al. (2001), who also placed surface EMG electrodes on the OE, and observed an increase in the activity of the right OE with an increase in the exertion level during the left and right trunk rotations. The OE is a contralateral axial rotator: Unilateral contraction of the OE leads to contralateral trunk rotation (Brown et al., 2011; Neumann, 2017; Ng et al., 2001) (Figure 1). Therefore, the EMG activity during the left trunk rotation could be attributed to the right OE activity, as contralateral trunk rotation was caused by unilateral contraction of the right OE. On the other hand, there are two possible EMG activity sources during the right trunk rotation; the first possible source is the antagonistic activity of the right OE (ipsilateral OE). Ng et al. (2001) explained that the antagonistic activity of the ipsilateral OE could be considered as an example of muscle co‐contraction, which could play an important role in stabilizing the spine during exertion at the expense of torque generated by the trunk rotation. The second possible EMG activity source during the right trunk rotation is the agonistic activity of the right OI (ipsilateral OI). The OE is the most superficial lateral abdominal muscle, and the OI is located immediately beneath the OE. The OE and OI are the most effective axial rotators of the trunk; however, the OE is a contralateral rotator, whereas the OI is an ipsilateral rotator (Brown et al., 2011; Neumann, 2017; Ng et al., 2001) (Figure 1). Hence, the OE on one side works synergistically with the OI on the opposite side during trunk rotation in a particular direction: The left OE (contralateral OE) and right OI (ipsilateral OI) work synergistically as agonists during the right trunk rotation. When considering the location of the OE and OI and their role in trunk rotation, surface EMG electrodes placed on the OE could possibly detect not only the antagonistic OE activity but also the agonistic OI activity during the right trunk rotation, which is the signal crosstalk between the OE and OI.

In the EMG amplitude at 100% 1RM, the percentage of the EMG amplitude during the right trunk rotation relative to the EMG amplitude during the left trunk rotation was 61 ± 30% (Figure 4). Ng et al. (2001) showed that during maximum voluntary left and right trunk rotations, the right OE was activated at approximately 48% and 35% of the largest possible amplitudes, respectively. Hence, the percentage of activation level during the right trunk rotation relative to that of during the left trunk rotation was approximately 73%. Andersson et al. (2002) and Juker et al. (1998) measured the activities of the OE during maximal voluntary trunk rotations using intramuscular EMG. Andersson et al. (2002) reported that the contralateral OE was activated at 42 ± 5% and the ipsilateral OE was activated at 20 ± 3%; therefore, the percentage of the ipsilateral OE to the contralateral OE activation level was approximately 48%. Juker et al. (1998) reported that the activation levels of the contralateral and ipsilateral OE were 52 ± 13% and 18 ± 8%, respectively; hence, the percentage of the ipsilateral OE to the contralateral OE activation level was approximately 35%. These results suggest that the percentage of the antagonistic activity of the ipsilateral OE to the agonistic activity of the contralateral OE was overestimated by surface EMG, which could be attributed to the signal crosstalk between the OE and OI.

Fuglevand et al. (1992) simulated the effect of interelectrode distance on the amplitudes of motor unit action potentials. When simulating the action potential of a large motor unit (innervating 2500 fibers with a territory diameter of 12.6 mm) using bipolar electrodes (electrode size = 0.04 cm^2^; interelectrode distance = 1.1 cm), the distance between the electrode and motor unit territory where the action potential decreased below the level of noise (i.e., the detection distance) was 2.7 cm. The electrode properties in the previous study were similar to those of the electrodes used in this study (electrode size = 0.05 cm^2^; interelectrode distance = 1.0 cm); therefore, the previously reported detection distance could be valuable in interpreting the findings of this study. The total thickness of the subcutaneous tissue and the OE overlying the OI at rest (Figure 2) was measured as 1.8 ± 0.3 cm, which was within the published detection distance of 2.7 cm. Consequently, the surface EMG sensor used in this study would have been able to detect the activity of the OI immediately beneath the OE.

During the left trunk rotation, increasing levels of exertion caused an obvious change in the color of the elasticity map of the right OE, but not in the color of the elasticity map of the right OI (Figure 5). When measuring the V_s_ by analyzing the elasticity map, the V_s_ of the right OE during the left trunk rotation significantly increased with increasing levels of exertion, except from 75% to 100% 1RM (Figure 6), which supports the finding that the EMG amplitude significantly increased as the level of exertion increased (Figure 4). Additionally, a significant correlation was found between the EMG amplitude and V_s_ of the OE, but not between the EMG amplitude and V_s_ of the OI in the left trunk rotation (Figure 7). Therefore, in the left trunk rotation, the EMG signals acquired using the surface EMG electrodes placed on the right OE seem to reflect the agonistic activity of the right OE. During the right trunk rotation, an obvious change in the color of the elasticity map with increasing levels of exertion was not observed in the right OE but was observed in the right OI (Figure 5). The V_s_ of the right OE in the right trunk rotation was significantly greater at 25%–100% 1RM than at rest (Figure 6), which was attributed to the antagonistic activity of the right OE. However, there were no significant differences in the V_s_ of the right OE during the right trunk rotation among 25%–100% 1RM, except between 25% and 75% 1RM (Figure 6). This result suggests that the antagonistic activity of the right OE during the right trunk rotation did not significantly increase with increasing levels of exertion. On the other hand, the V_s_ of the right OI during the right trunk rotation significantly increased with increasing levels of exertion, except between 50% and 75% 1RM (Figure 6). Therefore, the results for the right trunk rotation were considered inconsistent between the EMG amplitude and V_s_ of the OE, but consistent between the EMG amplitude and V_s_ of the OI. Moreover, during the right trunk rotation, significant correlation was not found between the EMG amplitude and V_s_ of the OE, but between the EMG amplitude and V_s_ of the OI (Figure 7). Hence, the surface EMG electrodes placed on the right OE could detect the agonistic activity of the right OI, as well as the antagonistic activity of the right OE during the right trunk rotation. More specifically, the increase in the EMG amplitude with increasing exertion level in the right trunk rotation was likely related more to signal crosstalk between the OE and OI than increased drive to the right OE that is the antagonist of the right trunk rotation.

Some limitations of this study need to be acknowledged. First, the total thickness of the subcutaneous tissue and the OE in the participants (1.8 ± 0.3 cm) were insufficient for entirely avoiding the influence of signal crosstalk between the OE and OI. In participants with a greater total thickness than those in this study, the OE activity could possibly be acquired using surface EMG electrodes without the confounding influence of the OI activity. Second, the quality of the measurements performed with ultrasound SWE was degraded by the depth and deformation of the OE and OI. In ultrasound SWE, shear waves are remotely generated by focusing an ultrasonic beam deep in the tissues. Thus, the layers of subcutaneous tissue and the OE above the OI interfere with the generation of shear waves within the OI. As a result, incomplete and unfilled color‐coded elasticity maps were often generated in the OI measurements (Figure 5), and the elasticity maps of the OI at rest could not be acquired for 5 of the 10 participants. Furthermore, the drastic deformation of the OE and OI (Figure 5), resulting from the exertion of force, made it difficult to acquire the elasticity maps of these muscles. With the drastic muscle deformation of the muscles, the examiner had to perform instantaneous fine adjustments of the handheld probe to maintain its positioning along the fascicle direction of the OE or OI. The time required for such fine adjustments was the longest during trunk rotation at 100% 1RM, resulting in V_s_ values that were considered underestimated. No significant increase in the V_s_ of the right OE from 75% to 100% 1RM in the left trunk rotation may be due to an underestimation of the V_s_ of the right OE at 100% 1RM. To ensure a sufficient acquisition time for the generation of the elasticity maps, the definition of the 1RM used in this study should be adapted in future studies; for example, changing the time to maintain the front‐facing position from 5 to 10 s. Third, the greater V_s_ of the antagonistic OE or OI at 25%–100% 1RM than at rest (Figure 6) could be attributed to two possible sources: (i) The antagonistic muscle activity that generates active force in the antagonist, and (ii) passive extension of the antagonist that generates passive muscle force in the antagonist. The reason for this third limitation was shown by Hug et al. (2015), who reviewed a series of previous studies that support the hypothesis that V_s_ acquired using ultrasound SWE is linearly related to both active and passive muscle forces. However, this was beyond the scope of this study to investigate the contribution of the two possible sources. Fourth, fatigue from performing six trunk rotations for each weight may have affected the EMG amplitude and V_s_. Neuromuscular fatigue would affect the EMG amplitude (Carr et al., 2016; Tachi et al., 2004; Walker et al., 2012) and V_s_ (Andonian et al., 2016; Morel et al., 2019; Siracusa et al., 2019). To minimize the confounding influence of fatigue, we administered the measurements of the EMG amplitude and V_s_ in a random order, strictly in a semi‐random order: EMG amplitude – V_s_ of the OE/ OI or V_s_ of the OE/OI – EMG amplitude. In addition, rest periods of at least 1 min were provided between the trunk rotations. Thus, the effects of neuromuscular fatigue should not drastically alter the findings of this study.

## 5 CONCLUSION

The findings on ultrasound SWE obtained in this study led to the conclusion that surface EMG electrodes placed on the OE appear to detect not only the activity of the OE, but also that of the underlying OI. Therefore, when acquiring the OE activity using surface EMG electrodes, it is necessary to consider the potentially confounding OI activity, that is, signal crosstalk between the OE and OI, especially during trunk rotations where the OE acts as the antagonist and the OI acts as the agonist.

## CONFLICT OF INTEREST

The authors declare that they have no conflicts of interest associated with this manuscript.
